# Advanced Topics and Smart Systems for Wireless Communications and Networks

**DOI:** 10.3390/s23156876

**Published:** 2023-08-03

**Authors:** Ali Mansour, Hadi Aggoune, Christophe Moy, Abbass Nasser, Muhammad Ayaz, Koffi-Clément Yao

**Affiliations:** 1 LABSTICC UMR CNRS 6285, ENSTA-Bretagne, 29806 Brest, France; abbass.nasser@ieee.org; 2 Artificial Intelligence and Sensing Technologies (AIST) Research Center, University of Tabuk, Tabuk 71491, Saudi Arabia; haggoune.sncs@ut.edu.sa (H.A.); ayazsharif@ut.edu.sa (M.A.); 3 CNRS, IETR-UMR 6164, Université de Rennes, 35000 Rennes, France; christophe.moy@univ-rennes1.fr; 4 Intelligent Computing and Communication Systems Lab (ICCS-Lab), Computer Science Department, American University of Culture and Education (AUCE), Beirut 1507, Lebanon; 5 LABSTICC UMR CNRS 6285, Université de Bretagne Occidentale, 29238 Brest, France; koffi-clement.yao@univ-brest.fr

## 1. Introduction

Telecommunication has shaped our civilization and fueled economic growth significantly throughout human history. Wireless communication technologies have continuously advanced and transformed the way people interact and communicate, from the development of the telegraph and radio broadcasting to the emergence of television and mobile phones. The telecommunications industry has grown quickly in recent years, becoming one of the major engines of our contemporary economy.

Although Maxwell, Hertz, and Marconi’s groundbreaking work in the late 19th century is where wireless communication first started, the area has made great strides and improvements in a relatively short amount of time. The telecommunications business has benefited from ongoing innovation and ingenuity, in contrast to the automotive sector, where engine technologies have largely remained untouched for more than a century. Scientists, researchers, and engineers have made a number of ground-breaking inventions in just the last two decades alone, stretching the bounds of what was previously thought to be feasible.

Radio, television, satellite communication, mobile phones, the Internet of Things (IoT), wireless networks, and many more fields have all become reliant on wireless telecommunication technologies. These innovations have impacted every aspect of our daily life, making smart cities, smart cars, biomedical engineering, security systems, and even global positioning satellite systems possible. It is impossible to exaggerate the significance of wireless communications and networks because they have become fundamental to our social interactions and have turned into important engines of economic growth.

The future of wireless technology looks bright but challenging. This Special Issue highlights new challenges, presents recent and future technologies, and discusses exciting potential applications related to wireless communications and networks. We are glad to announce the success of our Special Issue, as we received 11 contributions summarizing some of the hottest areas in wireless communications and related topics. These contributions are the hard work of 34 authors affiliated with 20 institutes from 11 countries located on the 5 continents (Australia, Czech, Egypt, France, Japan, KSA, Lebanon, Pakistan, Slovakia, Spain, and USA). We are very thankful for their contributions. And we would like to thank MDPI’s *Sensors* Editorial Board for its technical and logistical support.

## 2. Overview on Contributions

Mortada et al. consider the cognitive radio wireless sensor networks (CRSN) and how to cluster a CRSN, such that the cluster head (CH) performs Spectrum Sensing (SS), gathers the data, and sends it toward a central base station by adopting an ad hoc topology with an in-network data aggregation (IDA) capability [1]. The authors showed that the energy consumed by the data transmission decreases, while the total consumed energy of SS increases, since more CHs need to perform SS before transmitting.

In order to extend the coverage and enhance the quality of our wireless networks, many new technologies have been proposed, including cloud, fog, mist, and edge computing. In [2], the author focuses his contribution on these new technologies and considers several issues such as the IoT, the main security and privacy challenges, and countermeasures to mitigate the effect of these security issues.

Dynamic shared access provides flexible spectrum usage over a citizen broadband radio service (CBRS) band, which is governed by a spectrum access system (SAS). In SAS, the users are classified in three categories: incumbent access (IA) users, primary access license (PAL) users, and general authorized access (GAA) users. In [3], Abbass et al. propose a dynamic channel assignment algorithm for PAL and GAA users to maximize the transmission rate and at the same time minimize the total cost of GAA users accessing PAL-reserved channels.

Visible light communication (VLC) has become a promising secure, energy-efficient, and cost-effective technology for high-data-rate communications and an attractive complementary to conventional radio frequency (RF) communication. The power domain nonorthogonal multiple access (PD-NOMA) scheme is envisioned to address several challenges in VLC systems. In their review article [4], Sadat et al. introduce insights on some PD-NOMA VLC system constraints and challenges, such as power allocation, clipping effect, MIMO, and security, and provide open problems as well as possible directions for future research to pave the way for the implementation of PD-NOMA VLC systems.

Barua et al. present an adaptive modulation scheme for underwater acoustic (UA) communication systems, with the aim of increasing efficiency [5]. They propose an approach to attain a high data rate in UA communication, as UA channels vary very quickly along with environmental factors. A real-time orthogonal frequency-division multiplexing (OFDM)-based adaptive UA communication system is presented in this paper. Recent UA communication experiments carried out in the Canning River, Western Australia, verify the performance of the proposed adaptive UA OFDM system, and the experimental results confirm the superiority of the proposed adaptive scheme.

Several studies have shown that standard routing protocols cannot be implemented in vehicular ad hoc network (VANET). Because VANET communication links are broken very frequently, it is necessary to address the routing consistency of these highly dynamic networks. To enhance the overall network performance, Karim Kazi et al. propose a protocol named compacted area with effective links (CAEL) [6]. When compared to previous protocols, such as Dynamic Trilateral Enrollment (DyTE) and Reliable Group of Vehicles (RGoV), simulations demonstrate that CAEL can achieve an overall improvement in the performance of the network.

The emerging network function virtualization (NFV) technology transforms legacy-ardware-based network infrastructure into software-based virtualized networks in order to increase the flexibility and scalability of networks and at the same time reduce the time for the creation of new networks. However, the attack surface of the network increases, which requires the definition of a clear map of where attacks may happen. Naim et al. represent threats as misuse cases and propose countermeasures in the form of security patterns to build a security reference architecture (SRA) [7].

Salika et al. propose a new protocol called LoRaCog to introduce the concept of cognitive radio (CR) into the LoRa network, which enables access to a wider spectrum than LoRaWAN by using the underutilized spectrum and thus has better efficiency without impacting the end devices’ battery consumption [8]. Simulations show the flexibility in the system to utilize the available frequencies in an efficient and fair way. The results obtained reveal that a lower number of GWs is needed for LoRaCog compared to LoRaWAN to cover the same area.

In the contribution of Banaamah et al. [9], the authors explore intrusion detection methods implemented using deep learning, compare the performance of different deep learning methods, and identify the best method for implementing intrusion detection in IoT. The empirical results are analyzed and compared with the existing approaches, and the proposed method has a higher accuracy in comparison.

In wireless sensor networks, collected data should be gathered by various sink nodes. The data collection/processing time increases with the number of nodes, and frequent transmission collisions degrade spectrum efficiency. The research work of Zhou et al. investigates the relay communication for the over-the-air computation (AirComp) technique and studies a relay selection protocol [10]. This proposed approach both enhances the network lifetime and reduces computation errors.

Compared to conventional RF systems, radio-over-fiber (RoF) and millimeter-wave systems enjoy several advantages, such as a high optical transmission rate, light weight, low cost, and a wide frequency range up to 2 THz. Therefore, many recent studies have considere these technologies in various applications: wireless fidelity (WiFi) communications, radar applications, and cellular networks. In their paper [11], Termos et al. design and evaluate two experimental systems for a single and a simultaneous electro-optical semiconductor optical amplifier Mach–Zehnder interferometer (SOA-MZI) mixing system based on the differential modulation mode. The largest frequency range achieved during the experimental work was 118.5 GHz. Moreover, the peak data rate of the 128-QAM up-mixed signal could reach 70 GBit/s.

## 3. Conclusions

This Special Issue provides a comprehensive overview of the advancements in and potential of intelligent solutions in the field of wireless communication and networking systems. The 11 papers published in this issue cover various aspects of wireless communication and networking systems, including communication protocols, security considerations, and novel applications. The findings and insights presented in these papers underscore the significance of smart systems in addressing the challenges faced in this field.

The contributions of the authors showcase innovative methodologies, empirical evidence, and practical insights that demonstrate the effectiveness of smart systems in enhancing the performance, efficiency, security, and scalability of wireless networks. These findings serve as a valuable resource for researchers and practitioners in the field, inspiring further advancements and collaborations.

By harnessing the potential of smart systems, we can pave the way for more intelligent, reliable, and interconnected wireless networks. The research presented in this Special Issue provides a solid foundation for future endeavors in the real world, and serves as a catalyst for further exploration and innovation, leading to transformative applications and technological advancements.
